# Symmetry–simplicity, broken symmetry–complexity

**DOI:** 10.1098/rsfs.2022.0075

**Published:** 2023-04-14

**Authors:** David C. Krakauer

**Affiliations:** Santa Fe Institute, Santa Fe, NM 87501, USA

**Keywords:** symmetry, broken symmetry, complexity, information, causality, computation

## Abstract

Complex phenomena are made possible when: (i) fundamental physical symmetries are broken and (ii) from the set of broken symmetries historically selected ground states are applied to performing mechanical work and storing adaptive information. Over the course of several decades Philip Anderson enumerated several key principles that can follow from broken symmetry in complex systems. These include emergence, frustrated random functions, autonomy and generalized rigidity. I describe these as the four Anderson Principles all of which are preconditions for the emergence of evolved function. I summarize these ideas and discuss briefly recent extensions that engage with the related concept of functional symmetry breaking, inclusive of information, computation and causality.

## 1. Beyond symmetry

It is high time for me now to close the discussion of geometric symmetries dwelling in ornaments and crystals. The chief aim of this last lecture is to show the principle of symmetry at work in questions of physics and mathematics of a far more fundamental nature, and to rise from these and its previous applications to a final general statement of the principle itself.

—*Symmetry*. Herman Weyl.

Thus I was launched into the search for the ‘Aufbauprinzips’ of the everyday world. I admired Frohlich’s book on dielectric theory because it took the same point of view; I encountered it in my early work on ferroelectrics. Much more important, I came to see the crucial importance of the concept of broken symmetry, which has been a life-long interest. Broken symmetry is the clearest instance of the process of emergence which lies behind ‘More is different’.

— *A Career in Theoretical Physics*. Philip Anderson.

In his 1952 monograph *Symmetry* [1], Herman Weyl summarizes a powerful perspective on reality, drawing on mathematics as applied to the physical domain. Weyl proposes that physical science is ultimately that form of inquiry whose foundations lie in the analysis of symmetries. The profusion of symmetry-related concepts in physics, to include space–time symmetries, Noether’s theorem, gauge symmetries and super-symmetry, place the idea of invariance and its sequelae—universal laws—at the heart of the study of the non-living universe. The success of this endeavour is undisputed given the importance of symmetry in describing the structure of both conservative dynamical systems and equilibrium states of matter. Spanning everything from celestial mechanics at the largest scales, to the crystallographic point groups at the molecular scale, and unitary groups at the quantum scale. The principle of symmetry, and its manifestation through the fundamental symmetrical laws of motion, has provided the basis for the pursuit of a grand theory of everything, in which models of the universe seek to be assembled from the bottom up.

Growing out of the study of condensed matter, rather then particles and their interactions, Philip Anderson engaged in a life-long pursuit of a far more expansive ‘afbau’ programme, or set of construction principles, through which microscopic interactions are finally subsumed by their macroscopic products. In 1977, Anderson received the Nobel Prize in physics in recognition of his theoretical work on disordered systems and their applied value in engineering switching circuits. Anderson’s research into the development of the macrosopic structure of the universe culminated in his anti-reductionist-physics emergence manifesto, More is Different, in 1972 [2]. Over the course of two decades leading up to More is Different, and well into the 1980s and 1990s, Anderson sought to ground complex phenomena in the macroscopic laws of statistical physics. A part of his epistemic project was to contribute to the founding of the Santa Fe Institute in 1984, and he summarized several of his own institutional motivations in his paper ‘The eightfold way to the theory of complexity: a prologue’. First published in 1994 [3] the title is a homage to Murray Gell-Mann’s Eightfold Way for classifying the hadrons. Anderson’s interest was in accounting for common structures that are not predicted from the symmetrical fundamental laws of physics. The foundational concept behind all of his work, and arguably all complex phenomena, is broken symmetry.

The consequences of broken symmetry are multiple but of greatest interest to the study of complexity are what I shall call the four Anderson Principles—these include: (i) emergent properties arising through multiplicity or scaling, (ii) the importance of ‘frustrated random functions’, (iii) the requirement of autonomy and (iv) the property of generalized rigidity. Taken together the Anderson Principles, derived largely for equilibrium macroscopic states, provide necessary foundations for understanding the emergence of quasi-equilibrium structures initiated in the non-equilibrium regime through dissipative, or driven, dynamics. The Anderson Principles fall short of a description of adaptive function, which lies beyond any theory limited to equilibrium structure, but need be components of any such theory.

In the following sections, I summarize these ideas, and where possible, extend them to include more recent results and phenomena that apply more directly to considerations of adaptive structure and function.

### 1.1. Broken symmetry: creating a space beyond physics

Anderson introduces the idea of broken symmetry at the start of his article ‘More is different’ by describing the scale-dependent limitations of symmetrical physical laws for explaining common macromolecular structures. The example he selects is the pyramidal inversion of amines. A single molecule of ammonia (NH_3_) is said to possess a pyramidal structure and flips or quantum tunnels between two states—up and down—at around 30 billion times a second. The consequence of this process is that the stationary distribution of ammonia is a mixture of two mutually invertible pyramids. The low tunnelling barrier between two configurations of ammonia implies that ‘the state of the system, if it is to be stationary, must always have the same symmetry as the laws of motion which govern it’. However, when one considers slightly larger molecules like phosphine (PH_3_), the inversion rate is at least an order of magnitude slower, and phosphorus trifluoride (PF_3_) which is yet more massive, at least as slow again. Once we reach biologically active molecules at the scale of even the simplest carbohydrates, the whole idea of parity symmetry breaks down. The stationary distribution of molecules is dominated by initial conditions rather than their laws of motion. In order to explain the distribution of observed structures we need an account that exceeds the parsimony of the fundamental laws. The additional parametrization beyond the description of the basic laws effectively ‘counts’ the broken symmetries, and through an appropriate measure of model complexity, provides evidence of emergence.

In a recent review, Buhse *et al.* [4] expand this perspective to chiral molecules out of equilibrium, and discuss several fundamental mechanisms of biased imperfect bifurcation associated with chiral symmetry breaking. In this case, racemic mixtures of chemical enantiomers (chemical mirror images) are tipped into stable homochiral solutions by environmental chiral processes, including fluid vortices and polarized light.

Anderson significantly extended his thinking on these topics in 1994 in ‘Some general thoughts about broken symmetry’ [5]. In this article, the ideas of autonomy and generalized rigidity are introduced which I shall cover in later sections. Here, I want to mention briefly the emphasis that Anderson places on phase transitions and broken symmetries as described by Landau’s theory of second order phase transitions [6]. It will be recalled that Landau showed that ‘Every transition from a crystal to a liquid or to a crystal of a different symmetry is associated with the disappearance or appearance of some elements of symmetry’. And that for second order phase transitions (sensu Ehrenfest) ‘continuous transitions (in the same sense that transitions between liquid and gas are continuous) connected with change of the symmetry of the body are absolutely impossible’. Anderson describes such phase transitions as movements towards lower symmetries. For example, the loss of rotational symmetry (isotropy) or translational symmetry (homogeneity) in condensed states of matter, such as some liquid crystals. For Anderson, the order parameter of a state of matter that changes through a phase transition should be thought of as a measure of the degree of broken symmetry, which in Landau theory is typically the degree of increased order. Moreover, the order parameter should be derived from an understanding of the symmetrical laws of motion. In this way, we see very precisely the degree to which the fundamental laws and emergent structure deviate from one another.

I suspect that the 1994 paper is unintentionally responsible for many confused statements where emergence and phase transitions have been treated as synonymous. Firstly, as Anderson is quick to point out, his ideas do not pertain to all phase transitions, such as the best known case, the transition from a liquid to a gas that is symmetry preserving. And in the case of a transition to the condensed broken symmetry state (increased order), it is precisely their potential for supporting numerous further broken symmetries (increased disorder) that make them so important for living systems. As he writes in More is Different, ‘Symmetrical as it is, a crystal is less symmetrical than perfect homogeneity’ and ‘This type of “information-bearing-crystalinity” seems to be essential to life’. Which latter point is of course a tribute to Schrödinger’s aperiodic crystal conjecture for inheritance in *What is Life?* [7].

#### 1.1.1. Emergence

The idea of emergence can be very simply described as the successful factoring out of the fundamental laws of physics without any loss of explanatory power. Anderson makes broken symmetry the primary mechanism for achieving this effect. Amino acids are homochiral despite the fact that their enantiomers are equally likely. And the same goes for whole cell chirality which has been explored in connection to spontaneous mirror symmetry breaking at the interconnected nucleic acid, protein and carbohydrate domain of protocells [8]. Physical laws are not sufficient to explain this variation. And at more inclusive levels of organization, these laws have little to say about action potentials coding for muscle contraction, the effect of bugs in Lisp code, why some food tastes bitter, or what makes a Wallace Stevens poem work. All of these are candidates for phenomena where the laws governing the basic structure of matter do not bear in non-trivial ways on the understanding of phenomena.

In ‘More is different’ Anderson presents a table with two columns, *X* and *Y*. Under *X* he includes a list of sciences that obey the laws of the sciences in column *Y*. For example, *X* (= chemistry) obeys *Y* (= many body physics), and *X* (= cell biology) obeys *Y* (= molecular biology). The point is that ‘obey’ and ‘determine’ are not synonyms. We might say that the difference between obey and determine are statements about the number of symmetries that have been broken. An operational definition of reductionism is the limiting case where the symmetries of the laws of physics determine the empirical distribution of observables. Under reductionism the meaning of obey and determine becomes identical. In the study of complexity we are pursuing effective theories that are determinate for quantities of interest and many of these theories bear little resemblance to the physics that they obey.

At a certain point the symmetries become so peripheral to a macroscopic behaviour that even the idea of broken symmetry loses its moorings. Such that, ‘At some point we have to stop talking about decreasing symmetry and start calling it increasing complication. Thus, with increasing complication at each stage, we go on up the hierarchy of the sciences’. And it is not only a question of accumulating free parameters but of developing whole new conceptual structures. Kauffman’s work on gene control circuits, or what we would now call regulatory networks, was pioneering in this sense by introducing a focus on minimal rule systems capable of producing stable differentiated states. It is the world of emergent rules and not fundamental laws that exemplify the complex domain [9].

I find the work of Laughlin *et al.* [10] a very compelling extension of the ideas of Anderson, particularly their presentation of mesoscale organization in ‘The middle way’. Building up beyond broken symmetries, they introduce the idea of ‘protected states’, which are states stable against small perturbations of the underlying equations of motion. Hence, ‘superfluidity, ferromagnetism, metallic conduction, hydrodynamics, and so forth are “protected” properties of matter—generic behaviour that is reliably the same one system to the next, regardless of details’. In the case of phosphorus trifluoride, protection against tunnelling is provided by properties of the chemical potential and the molecular wave function. In the case of a large macromolecule like DNA, protection is achieved through a very complicated, evolved suite of error-correction mechanisms, that are imposed on the primary information-transmitting structures. Emergence at living scales is perhaps best thought of in terms of mechanisms of protection, or robustness [11], against large perturbations enabling the ‘effective’ equations of motion regulating biomolecules and their aggregates. With these screening-off mechanisms in place, we are permitted, for example in the case of disease monitoring, to substitute what would be the prohibitively cumbersome framework of structural biology required to accurately describe RNA and DNA, with vastly simplified strings comprising four letter alphabets.

#### 1.1.2. Frustrated random functions

In 1983, Anderson wrote a somewhat neglected paper, ‘Suggested model for prebiotic evolution: the use of chaos’ [12]. The paper has not proven to be particularly illuminating about the origin of life but it does include several important insights into the challenges presented by the theory of dissipative structures, and emphasizes the keystone function of frozen fluctuations as affordances for adaptive phenomena.

The theory of dissipative structures was introduced by Prigogine in a 1978 paper, ‘Time, structure and fluctuations’ [13], that summarized certain new ideas from non-equilibrium thermodynamics as applied to life developed over the previous decade at the University of Brussels. The bare bones of the theory is that non-equilibrium structures provide an important source of proto-order in living systems as for example in the vortices generated in a Bernard convection cell. Prigogine argued that these regularities represent a blind spot in Boltzmann’s equilibrium order principle. In a driven system, coherent states (dissipative structures) can emerge spontaneously and become stabilized through the exchange of energy with the outside world. A feature of these structures is that they remain very sensitive to ‘global features which characterize the environment of chemical systems, such as their size and form, the boundary conditions imposed on their surfaces’ and so forth. For Anderson this sensitivity proved to be an insurmountable bug (which I shall discuss in the following sections on autonomy and rigidity), such that without some means for equilibrium structures to condense out of the dynamics, the power of stationary broken symmetries could not be harnessed to store information reliably.

Anderson presents the problem as one of reconciling the need for stability with the requirements of diversity. However many dissipative structures might come into existence in a driven system, most will be transient, and the observed forms will be highly limited by their instability. The problem is that while ‘It is equally easy to set up a process with a stable result but only at the expense of losing a crucial degree of diversity’. Because stability sorts out a very small ‘viable’ set from a large number of candidate structures, ‘chaos is a precondition: we require a survival probability that is a fixed but chaotic function of the molecular composition’. Anderson suggests that the most compelling candidate system from equilibrium statistical mechanics effectively coupling stability with ongoing sources of randomness is the spin glass. That is a system with the spin Hamiltonian,

1.1 $$H=-\sum_{i,j}J_{ij}S_{i}S_{j},$$

with the special property that the exchange integrals *J*_*ij*_ are random functions of the variables (*i*, *j*). The most immediate implication of this randomness is that cycles in the graph defined by the matrix *J* imply that not all local spin-interactions can be satisfied. This gives rise to the idea of ‘frustrated random functions’. Since the number of metastable solutions of the Hamiltonian increase exponentially in system size, and the relaxation time between states is very slow, diversity can be preserved alongside quasi-stationarity.

Anderson deploys the frustrated random functions of the spin glass to define a simple model of sequence growth, splitting and complementary base-pairing. In the presence of a relatively weak random field (the environmental bias imposed on the spin states of the system), this is capable of supporting a diverse set of stable sequences. A subsequent and more compelling application of spin glasses to adaptive phenomena was provided by Hopfield [14] to explain content addressable memory. Hopfield makes the crucial observation that the degenerate ground states provided by the frustrated random interactions are capable of storing sequences of bits. Hence broken symmetry intrinsic to the system enables storage of persistent sources of diversity present in its environment.

In his 1987 paper, ‘Spin glass Hamiltonians: a bridge between biology, statistical mechanics, and computer science’ [15], Anderson emphasizes the phase transition of the Hamiltonian in the *N* → ∞ limit, where the system breaks ergodicity and different regions in the phase space are separated by energy barriers of order *N*^*p*^ where *p* < 1. The spin glass is the simplest equilibrium system which solves the obvious limitations of the scale-dependent sensitivity of dissipative structures by ‘freezing’ random fluctuations into stable condensed matter. This property gives rise directly to an essential empirical property of all living systems, and the third Anderson Principle, autonomy.

#### 1.1.3. Autonomy

Prigogine [13] highlights the scale-dependence of dissipative structures that arise from small fluctuations to an average state. And how these fluctuations grow to an amplitude constrained by system length. Many of the more compelling examples possessing these features are drawn from fluid dynamics, to include the above convection, but also cyclones, hurricanes and the Belousov–Zhabotinsky reaction. More recently researchers have made the claim that all living structures are dissipative structures, or ‘Bio-analogue dissipative structures’ (see [16] for a recent review). While this is a very interesting idea, and undoubtedly some part of an answer to the origin of adaptive form, Anderson extended his critique from sensitivity to include a far more subtle and functionally important deficiency—a lack of autonomy [5]. As Anderson states the case, ‘By autonomy of a structure I mean that its space or time structure should not be predetermined in terms of the scale of the external boundary condition’. And autonomy is an overwhelming feature of all complex forms of life—cells in a flea are not smaller than those in an elephant, the respiratory complex is the same structure and scale in all forms of life, and the length of genes does not depend on the size of cell or organism in which they reside. This list could be extended more or less ad infinitum.

It is very difficult to generate truly ‘autonomous’ dissipative structures at scales above simple physical devices, for example a laser, whose functions are not far removed from physical laws. The original formulation of a dissipative structure is in Alan Turing’s theory of morphogenesis [17] which in addition to showing an extreme sensitivity to domain size, suffers from a ‘fine-tuning’ problem, whereby stable standing waves require rather careful tuning of the diffusivity of morphogens (see analysis and possible solutions in [18] and [19]). There have been efforts to apply reaction–diffusion systems to biological phenomena, such as patterning in fish skin, but as researchers have pointed out, some additional source of non-dissipative memory is required to overcome the intrinsic spatial wavelengths induced by singular parameter values [20].

Anderson argued that dissipative dynamics play an essential early role in breaking symmetries and that these are subsequently condensed into quasi-equilibrium patterns. In the domain of morphogenesis, this corresponds to fluctuation-driven instabilities yielding Turing patterns that are after some delay consolidated by genetic regulatory networks (longer term memory) [21].

What has received far less attention is the crucial kinematic role of morphogenetic patterns. Even if dissipative structures can be stabilized and made scale-independent while retaining growth sensitivity, this falls far short of a full volumetric pattern performing any kind of useful mechanical work. This leads to Anderson’s fourth principle, generalized rigidity.

#### 1.1.4. Generalized rigidity

Rigidity is often defined as the ability of a solid to withstand deformation when it undergoes mechanical stress. The importance of rigidity was first explored by James Clerk Maxwell in relation to the propagation of forces through a sequence of rigid bodies in his paper, ‘On reciprocal figures and diagrams of forces’ [22]. Nearly all of our intuitions about classical mechanics, from billiard balls, springs and levers, through to animal locomotion, make use of the concept of rigidity. After all, flexible parts do not make for reliable mechanical clocks.

Anderson is interested in all the rigid interactions required to enable a control parameter (such as temperature) to transmit their forces through a stressed system in order to reveal themselves in an appropriate order parameter (such as spin-angle). Any non-trivial mechanism must possess a generalized rigidity with, ‘(1) … internal degrees of freedom which are not predetermined; (2) having a freedom which may be stably manipulated and which can exert action at a distance’ [5].

While rigidity is obviously important for any kinematic features of an organism or machine, it is equally important for information processing. An obvious example are transistors within an integrated circuit. These are semiconductors that transmit current through pairs of terminals placed into circuits of fixed topology. If integrated circuits could not reliably transmit current but were flexible (in this case leaking current at significant rate) computation would not be possible. And the same is true for the propagation of electrochemical signals throughout the nervous system. A flexible reflex would be little more than a gentle maladaptive spasm. There is a good functional reason why we bother to describe a connectome—the connections among neurons are functionally informative because they reveal information about the preferred propagation of electrochemical signals.

And a key to the reliable construction of rigid systems, as Anderson states in relation to the independence of internal degrees of freedom, is autonomous parts. For example, molecular motors in the cell make use of Brownian ratchets to propagate forces through rigid interactions, that ‘involves biasing, or rectifying, the otherwise random Brownian movements by using short-range attractive forces to trap favourable fluctuations’. And these drive reactions forward ‘in monomer-sized steps, using the energy that binds the monomers together to ratchet the fluctuations of the filament and the load’ [23].

More fundamentally, generalized rigidity is the physical precondition for causality. Hume defined causality in terms of reliable causes and effects, ‘We may define a cause to be an object followed by another, and where all the objects, similar to the first, are followed by objects similar to the second. Or, in other words, where, if the first object had not been, the second never had existed’. Adopting the language of Halpern [24], rigidity renders sensible both the idea of a regularity (A then B) and the counterfactual (not A then not B).

**Figure 1.**
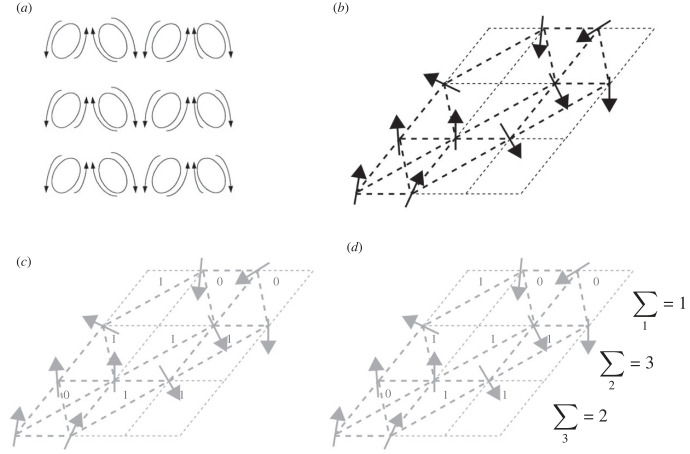
The four ‘Anderson Principles’ sequentially realizing an information processing mechanism. (*a*) Starting with a dynamical dissipative structure (e.g. diffusion driven instabilities in a Turing system) promoting patterns of inhomogeneity with a small number of ‘self-organized’ effective dimensions, that are ‘frozen’ or condensed into a frustrated equilibrium structure (*b*) (e.g. morphogen induced persistent patterns of gene expression). Stable broken symmetries are able to act as information storing disordered states at an ‘autonomous’ length scale (*c*) and used to propagate (e.g. through direct sequence binding or electrochemical signalling) information reliably across the structure (generalized rigidity) to perform a simple computation (*d*). (Panels (*b*–*d*) modified from https://en.wikipedia.org/wiki/Spin_glass.)

## 2. Coda: symmetries, information and evolution

The four Anderson Principles, as I have labelled them for expository purposes, expand our understanding of the crucial role that symmetry and broken symmetry play in complex systems. I find that these principles are a corrective to much questionable thinking on the relationship of phase transitions to emergence, dissipative structures to adaptive traits and patterning to both mechanical and computational work. I sometimes think of them as a minimal checklist to ensure that any proposed mechanism might become more than an intriguing pattern. The four principles are illustrated in an implicational order in figure 1.

Two of the areas that Anderson did not explore deeply and that follow naturally from his inquiries into emergence are information theory and evolution. One is arguably the most effective theoretical foundation in any effort to measure complication (beyond simple broken symmetry), and the other, the pinnacle of all domains in which broken symmetry is the foundational concept—more often described as ‘frozen accidents’.

Information entropy is after all a counting statistic for broken symmetries (every yes/no answer in 20 questions reduces the cardinality of a permutation group). And through mutation-drift-selection parsimoniously reconstructed phylogenies place traits at root positions to minimize broken symmetries (the Hennig auxiliary principle). The method of maximum entropy assumes symmetry in order to place on it constraints or broken symmetries that are defensible by fit to data [25]. And complexity more generally is probably best thought of as a minimal history consistent with observable variation [26]—in other words, the most symmetric (e.g. parallizable) process that can turn random inputs into regular outputs [27].

## Data Availability

This article has no additional data.
